# Interobserver and intraobserver reliability of a new prognostic classification for foot and ankle charcot arthropathy

**DOI:** 10.1186/s13018-025-06093-4

**Published:** 2025-08-07

**Authors:** Mohamed Abdelaziz Elghazy, Samer Ali, Ahmed El-Hawary, Hani El-Mowafi, Yasser Roshdy Kandil

**Affiliations:** Orthopedic Department, Faculty of Medicine, Mansoura University, Mansoura, Egypt

**Keywords:** Charcot Arthropathy, Classification Reliability, Foot and Ankle Charcot, Diabetes

## Abstract

**Background:**

Recently, a new prognostic classification for foot and ankle Charcot arthropathy (Mansoura Classification) was published. The aim of this study was to evaluate the interobserver and intraobserver reliability of Mansoura classification for foot and ankle Charcot arthropathy.

**Methods:**

Mansoura classification for foot and ankle Charcot arthropathy was presented to orthopedic surgeons (raters) who participated at Mansoura International Foot and Ankle Course. Thereafter, twenty cases of foot and ankle Charcot were presented to the raters who were asked to rate each case according to the classification. Three weeks after the initial evaluation, six raters from a single institution repeated the rating of the cases. Both interobserver and intraobserver reliability were assessed using Kappa statistics.

**Results:**

Sixty-one raters completed the evaluation of the twenty cases of foot and ankle Charcot. Their interobserver reliability was moderate (Kappa = 0.5). Intraobserver reliability was excellent for six raters (range, 0.81–0.93). Further analysis of the interobserver reliability of the former six raters who were specialized in foot and ankle surgery showed substantial reliability; Kappa = 0.67.

**Conclusion:**

Mansoura classification for foot and ankle Charcot has acceptable reliability which is comparable to other classifications. It could be promising in evaluating the severity and guiding the management of such cases.

**Level of evidence:**

Level IV case series.

## Background

Foot and ankle Charcot arthropathy is a devastating condition that can occur in up to 12% of patients with diabetes mellitus [1]. It can affect any region of the foot and ankle including the ankle, hindfoot, midfoot and forefoot joints. The management of diabetic Charcot arthropathy is challenging, and can lead to several complications including below knee amputation. An ideal classification system for Charcot arthropathy should include all the regions in foot and ankle, be able to guide the treatment and prognosis based on the severity of cases, and have a good reliability to ensure the consistency of measurements.

Several classification systems have been described for foot and ankle Charcot arthropathy [2–9]. The majority of the classification systems are based on either the developmental stages such as Eichenholtz classification and Armstrong and Lavery classification [4–6] or the anatomical regions affected based on radiographic evaluation without considering the clinical presentation such as Brodsky et al. and Sanders and Frykberg classifications [2, 7, 9], or did not include all the anatomical regions in the foot and ankle that could be affected by Charcot arthropathy [3, 8, 10]. 

A recent classification (Mansoura Classification) was published in an attempt to guide both treatment and prognosis of diabetic Charcot arthropathy [11]. Mansoura classification is applied mainly to post-acute Charcot. This new classification accounts for the number of affected regions in the whole foot and ankle and the clinical presentation as well as the associated complications. It considers the presence of deformity, instability and ulceration. The classification has two main types depending on the number of affected regions within the foot and ankle. Type I includes only one affected region and type II includes more than one affected region. Furthermore, each type is divided into four stages: (stage A: stable with no deformity, stage B: stable with deformity, stage C: unstable, stage D: associated mechanical ulcer). This classification guided the treatment and prognosis for each stage and it has the potential to predict amputation which occurred only in type IID.

Although the reliability of four classifications was evaluated in two prior studies [12, 13], the reliability of Mansoura classification has not been previously evaluated. The aim of this study was to evaluate both the interobserver and intraobserver reliability of Mansoura classification for foot and ankle Charcot arthropathy.

## Methods

This study received Institutional Review Board (IRB) approval from Faculty of Medicine, Mansoura University.. After institutional IRB approval, medical records were reviewed and the plain radiographs of twenty different cases of Charcot arthropathy of foot and ankle were captured and deidentified. The number of cases was chosen based on the previous reliability study performed by Schon et al. [13] The review process was conducted and the cases were selected to represent all the types and stages of foot and ankle Charcot as described in Mansoura classification. Subsequently, the selected cases were reviewed and approved by a foot and ankle consultant. Exclusion criteria were non diabetic Charcot arthropathy, previous surgery to the foot or ankle, and incomplete X-rays.

**Fig. 1:**
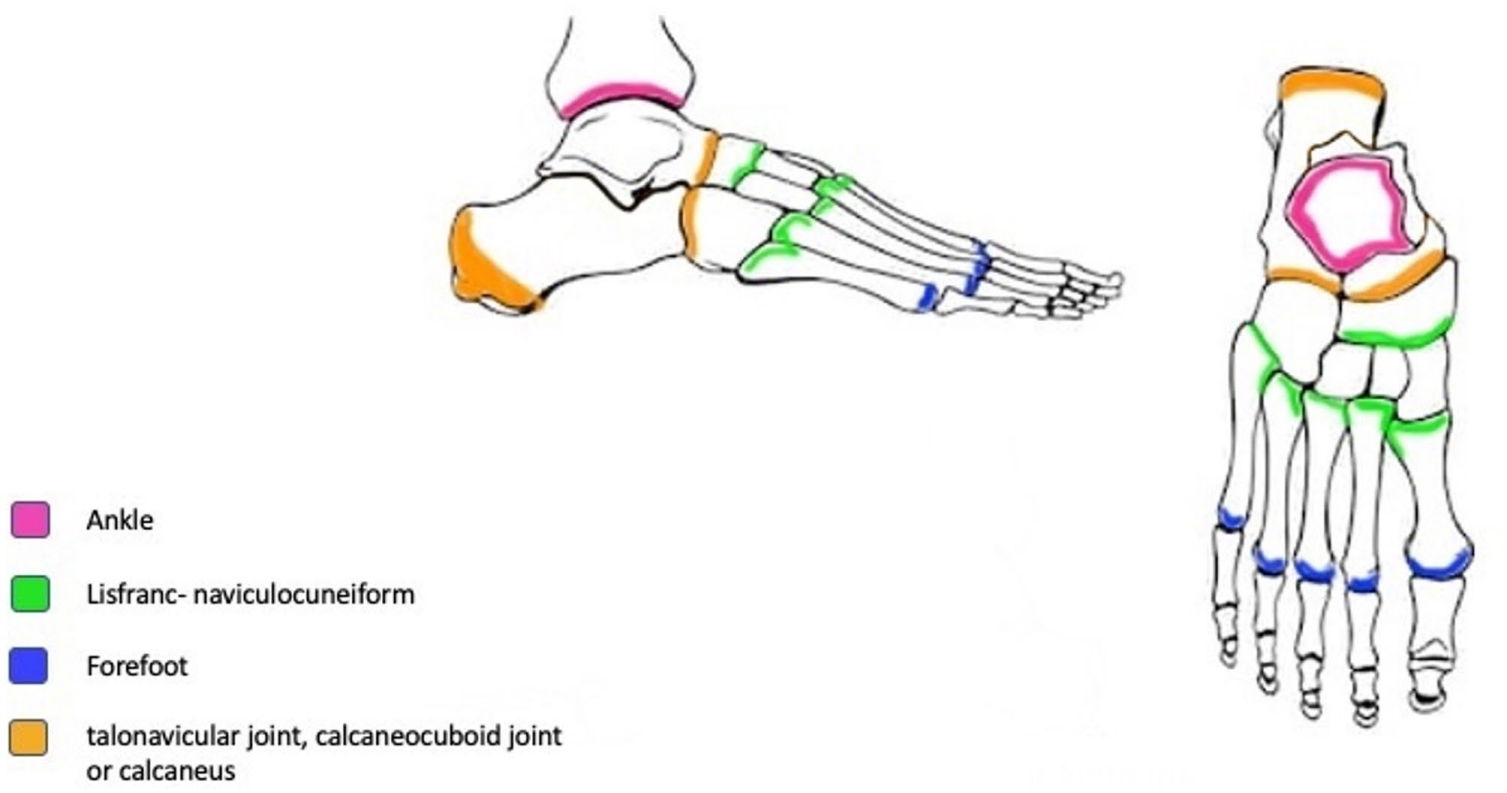
Regions affected in Type I Charcot

**Fig. 2:**
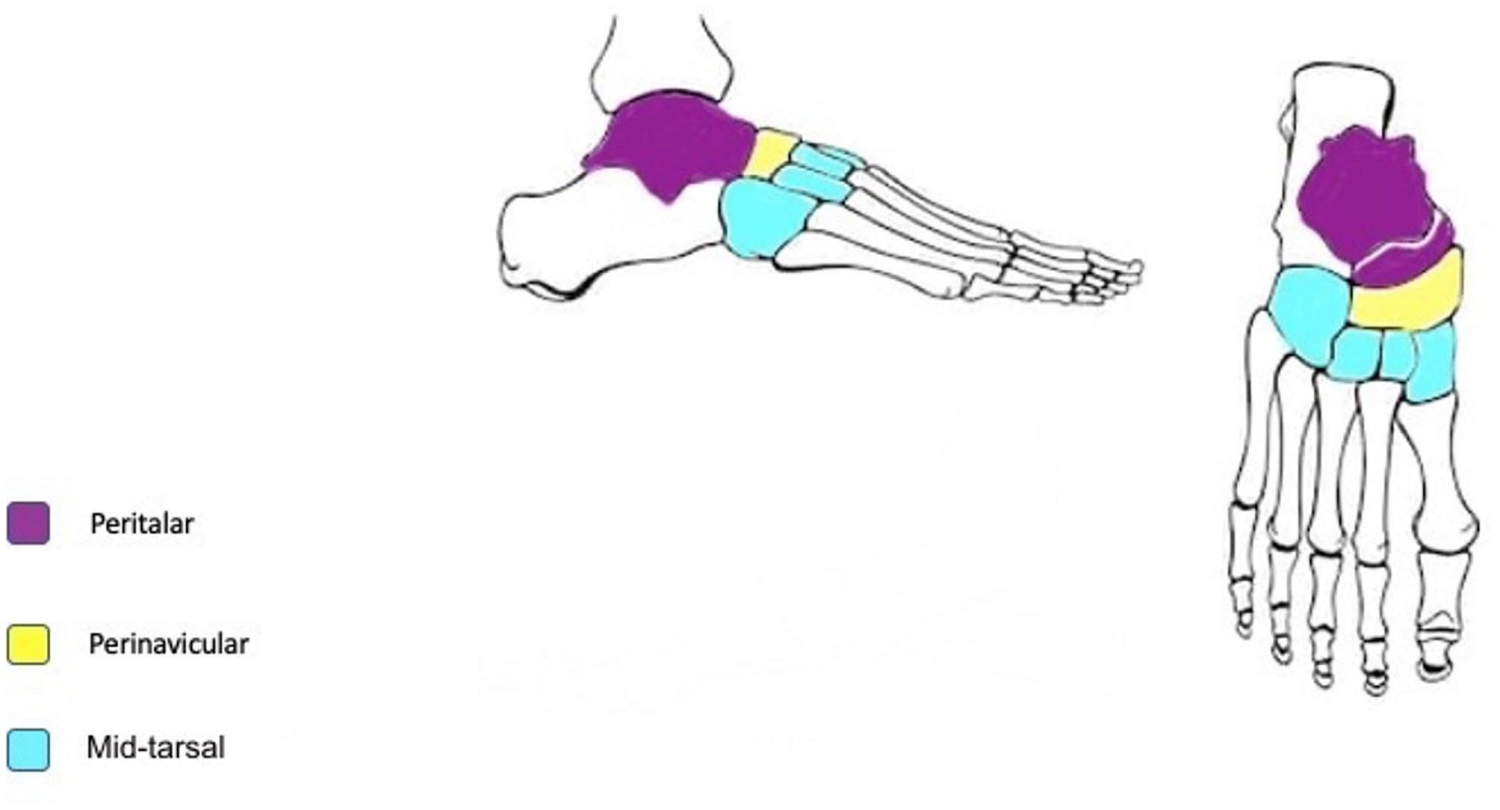
Regions affected in Type II Charcot

Mansoura classification for foot and ankle Charcot arthropathy was presented and explained to the participants (raters) at Mansoura International Foot and Ankle Course (MIFAC) over a 15-minute onsite lecture during one of the course sessions. Then the raters were given the classification table (Table 1) and colored printed copies of the classification diagrams (Figs. 1 and 2) [11]. They also received a sheet to rate each case according to the classification, and to list their current job role and years of experience which were classified as less than 5 years, 5–9 years, 10–14 years, 15–20 years and more than 20 years. Participation was voluntary. Additionally, the evaluation sheets did not contain names, ages or countries of the raters to keep the data deidentified and avoid bias.

**Table 1:** Mansoura classification for foot and ankle Charcot

Type	Description
IA	Single region, stable, no deformity
IB	Single region, stable, with deformity
IC	Single region, unstable with or without deformity
ID	Single region, stable or unstable, with deformity and mechanical ulcer
IIA	More than one region, stable, no deformity
IIB	More than one region, stable, with deformity
IIC	More than one region, unstable, with or without deformity
IID	More than one region, stable or unstable, with deformity and mechanical ulcer

Thereafter, these twenty cases of foot and ankle Charcot were presented to the raters in a random order, case by case via a PowerPoint presentation. The clinical information for each case was presented to the raters including the clinical examination whether the foot and ankle were stable or not, clinical pictures that showed the presence or absence of ulcers, and radiographs that showed both the anatomic regions affected and the deformity; if present. Then the raters were asked to rate each case according to the classification. All raters were asked not to share their thoughts with others during rating the classification, and the sheets were collected from all the raters after completion of the ratings.

Furthermore, six raters from a single institution were asked to repeat the classification of the cases. The cases were sent to them electronically at least three weeks after the initial evaluation in the course and non-simultaneously to minimize potential interaction. They were two clinical foot and ankle fellows, two foot and ankle specialists and two foot and ankle consultants. The choice of these six raters aimed to assess both the intraobserver reliability and the interobserver reliability among foot and ankle surgeons.

Cohen’s Kappa statistics were used to evaluate both interobserver and intraobserver reliability using STATA 14.2 program, and the results were interpreted as reported by Landis and Koch [14] as follows: 0.00–0.20 Slight, 0.21–0.40 Fair, 0.41–0.60 Moderate, 0.61–0.80 Substantial; and 0.81–1.00 Excellent. The *P* value was considered significant if < 0.05.

## Results

The evaluation sheets were collected from 63 raters, but two of them did not complete the rating of all the cases so they were excluded. Sixty-one raters with different levels of experience completed the evaluation of the twenty cases of foot and ankle Charcot. The raters were 7 orthopedic surgery residents, 19 fellows, 11 consultants, 7 assistant professors and 17 professors of orthopedic surgery. The twenty cases represented all eight stages of the classification as shown in Table 2. The interobserver reliability for all 61 raters was moderate (Kappa = 0.5) and the *P* value was < 0.0001, Further analysis according to the level of experience based on the years of practice of orthopedic surgery showed similar moderate interobserver reliability (range, 0.4–0.57), and the P values were < 0.0001 for all levels. (Table 3)

**Table 2:** Number of cases included in the evaluation based on each stage of the classification

Classification stage	Number of cases included in the presentation
IA	1
IB	2
IC	3
ID	3
IIA	1
IIB	2
IIC	4
IID	4

**Table 3:** Interobserver reliability for each level of experience

Years of experience	Number of raters	Interobserver reliability^*^ (Cohen’s Kappa)	95% Confidence Interval	Standard Error
less than 5 years	6	0.508	0.460–0.556	0.024
experience 5–9 years	19	0.572	0.557–0.587	0.008
experience 10–14 years	11	0.574	0.548-0.600	0.013
experience 15–20 years	8	0.402	0.366–0.438	0.019
experience more than 20 years	17	0.416	0.400-0.432	0.008

^*^P values for all results were < 0.0001

**Fig. 3:**
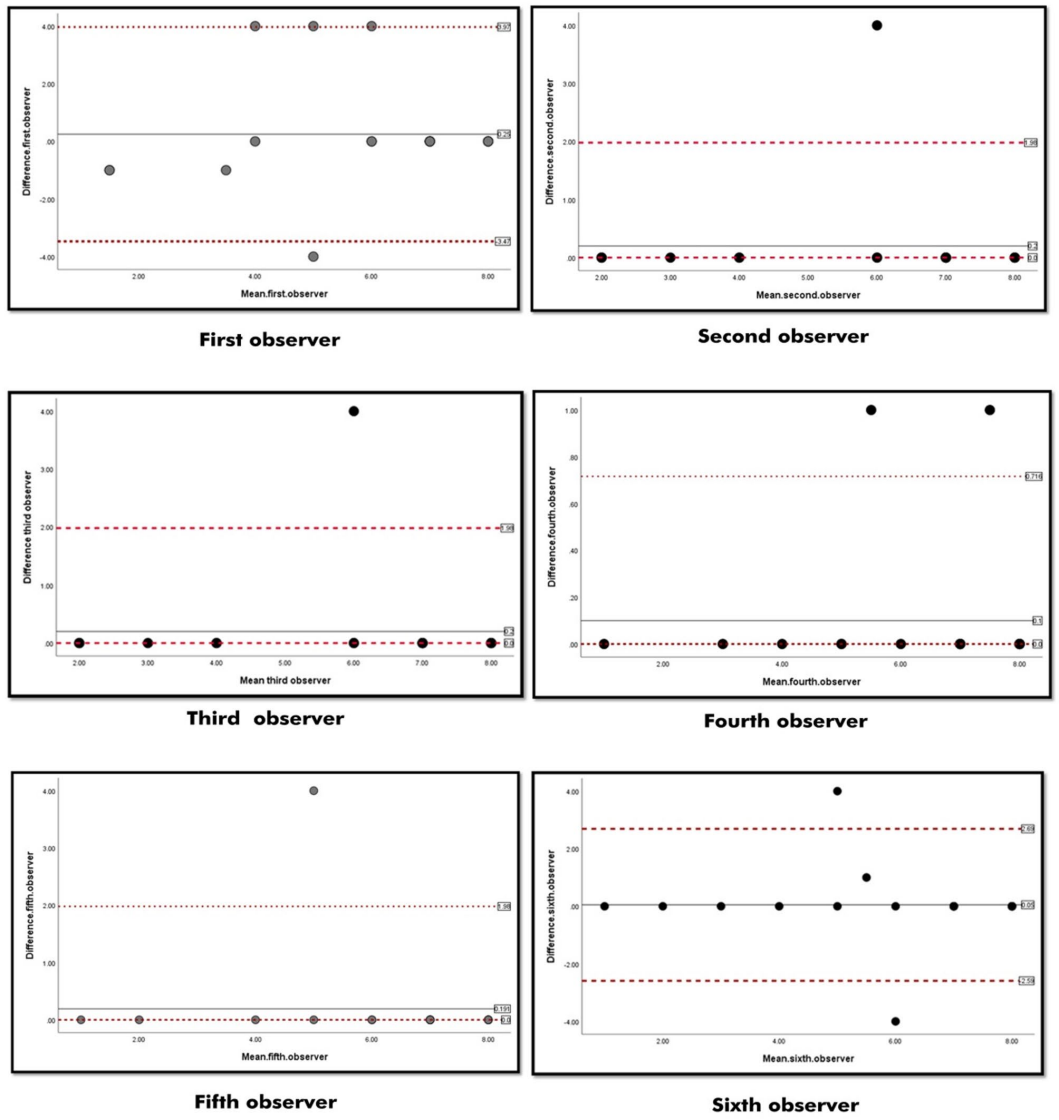
Bland-Altman plots for intraobserver reliability for 6 raters

Intraobserver reliability was excellent for all six raters (Kappa range, 0.81–0.93), with excellent percent of agreement (Table 4). Bland-Altman plots for intraobserver reliability showed no systematic differences between the measurements (Fig. 3). Further analysis of the interobserver reliability of the former six raters who were specialized in foot and ankle surgery showed substantial reliability; Kappa = 0.67 and *P* < 0.0001.

**Table 4:** Intraobserver reliability for 6 foot and ankle surgeons

Rater	Percent of Agreement	Cohen’s Kappa^*^	95% Confidence Interval	Standard Error
1	90.00%	0.870	0.644–1.09	0.115
2	95.00%	0.936	0.715–1.15	0.112
3	95.00%	0.935	0.716–1.15	0.112
4	90.00%	0.869	0.642–1.09	0.116
5	95.00%	0.933	0.705–1.16	0.116
6	85.00%	0.811	0.605–1.02	0.105

^*^P values for all observers were < 0.0001

## Discussion

Although several classification systems have been described for foot and ankle Charcot arthropathy, most of them are descriptive and could not guide treatment or prognosis. The present study aimed to evaluate both the interobserver and intraobserver reliability of a new classification system that considers both clinical and radiological information and has the potential to guide treatment and prognosis. Our study showed that Mansoura classification had excellent intraobserver reliability, substantial interobserver reliability for foot and ankle surgeons and moderate interobserver reliability for orthopedic surgeons with different subspecialties.

The reliability of three commonly used classifications for foot and ankle Charcot was evaluated in the study performed by Wukich et al. [12]. They reported excellent interobserver reliability for the Sanders and Frykberg classification, good to excellent for the Modified Brodsky classification and moderate to good reliability for the Eichenholtz classification. Although the results of the interobserver reliability for these three classifications are slightly higher than our results for Mansoura classification, it could be attributed to the study design itself. In the study of Wukich et al. [12], the statistical analysis was performed using Intraclass oCorrelation Coefficients (ICC). In addition to that, the raters were four board certified foot and ankle surgeons and one senior musculoskeletal radiologist, which could explain the higher interobserver reliability. In our study, the 61 raters were the participants in an international foot and ankle course with different backgrounds. The raters had different experience levels with different academic and clinical backgrounds. They were a mixture of orthopedic surgery residents, general orthopedic surgeons, foot and ankle specialists, and other orthopedic subspecialty surgeons who were aiming to acquire more knowledge in the field of foot and ankle surgery. Although that might explain the lower interobserver reliability among all 61 raters, it is more relevant to the clinical perspectives where the classification could be used at different levels of experience. In addition to that, further analysis of the interobserver reliability among six foot and ankle surgeons revealed higher interobserver reliability (Kappa = 0.67). These results match the reliability results obtained by Schon et al. [13] who evaluated the interobserver and intraobserver reliability of Charcot midtarsal classification. They reported higher interobserver reliability among foot and ankle fellows and lower interobserver reliability among attending orthopedic surgeons.

Another factor in the study done by Wukich et al. which might have contributed to the higher interobserver reliability is that they excluded any patient with infection or ulcer from the evaluation cases although it was shown that foot ulcers are present in up to 27% of cases of foot and ankle Charcot [11]. In our study, the twenty cases used for evaluation included cases with foot ulcers in different regions. Additionally, Mansoura classification has larger number of types which could potentially result in lower interobserver reliability when compared to classifications with lower number of types.

On the other hand, the intraobserver reliability of our classification was excellent (Kappa range 0.81 to 0.93), which matches the intraobserver reliability of Sanders and Frykberg, Modified Brodsky and Eichenholtz classifications [12]. Although the intraobserver reliability in our study was done based on lower number of raters as compared to interobserver reliability, our number of intraobserver evaluations (6 raters) was larger than the study done by Wukich et al. (5 raters).

The previous classifications have their own limitations. Although both Sanders and Frykberg classification and the modified Brodsky classification considered all the anatomic regions in the foot and ankle that could be affected by Charcot arthropathy, they are mainly descriptive and dependent on radiographs only without considering clinical information such as the presence of ulcers, deformity or instability. Additionally, there is no severity rating associated with these classifications. Furthermore, these two classifications could not guide either the treatment or the prognosis, as they only described which anatomic region is affected without considering the clinical presentation, and no mention of what to do in each stage. Additionally, the Eichenholtz classification does not represent the symptoms of Charcot arthropathy and does not cover the whole spectrum of the Charcot foot, and therefore cannot be used to guide the treatment or the prognosis.

Another classification for midtarsal Charcot that showed high reliability was the Schon classification. However, it was dependent solely on radiographs, and the ankle was not included in this classification which constituted up to 33% of cases of foot and ankle Charcot in a previous study [11]. It should be noted that Sella and Barrette were the first to combine both the anatomical and clinical descriptions, however they were limited to medial column only [3]. 

Mansoura classification incorporates both clinical and radiographic information, and has severity grading which increases as the stage increases from A to D. The clinical information includes the stability/instability and the presence or absence of ulcers. Instability evaluation is performed mainly through routine clinical examination. For example, ankle instability is determined through anterior drawer, varus and valgus stress tests, while midfoot instability is evaluated by applying the same principle through which the hindfoot is fixed with one hand and the forefoot is stressed by the other hand through both medial/lateral and dorsal/plantar movements to evaluate if midfoot instability is present. The ulcer component in stage D is the presence of mechanical ulcer secondary to the deformity, regardless of the ulcer size.

The radiographic information includes the regions affected and the presence or absence of deformity. Deformity evaluation on xray is done using the normal foot and ankle radiographic parameters to check if there is disturbance of these normal values that determine the presence of deformity like ankle varus/valgus, midfoot adduction/abduction, and medial arch collapse [15]. 

This new classification has the ability to guide treatment and prognosis in the clinical practice. Types 1 A and IIA can be managed conservatively as they are stable without deformity. All patients with types IC, ID, IIB, IIC, and IID require surgery to correct the deformity, achieve stability and offload the ulcer, if present. Type IB can be managed conservatively especially if the affected region is the medial Lisfranc joint. If type IB is localized to the ankle joint, it is better to perform surgery to correct the ankle deformity as it tends to progress. Patients with Type IID have increased risk for amputation [11].

Our study has some limitations. There is a potential interaction between the 61 raters during the evaluation of cases. However; stratification of the raters based on the level of experience showed similar reliability. Additionally, we performed a separate analysis for six foot and ankle surgeons which showed higher interobserver reliability. The second limitation is that the stability/instability information requires clinical examination, which is not applicable to allow all the raters to examine the same patients. Another point that could be considered a limitation is that we did not include radiologists in the evaluation. However, all the raters were orthopedic surgeons who would directly apply the classification in their practice for the management of foot and ankle Charcot. Additionally, the intraobserver reliability was evaluated based on six raters from a single institution and further analysis may be warranted to confirm the generalizability of these results. It should be noted that neither Mansoura classification nor any of the previously used classifications has been validated yet.

## Conclusion

Mansoura classification for foot and ankle Charcot arthropathy has excellent intraobserver reliability. Although the overall interobserver reliability among orthopedic surgeons was moderate and comparable to other classifications, the interobserver reliability for orthopedic surgeons who were specialized in foot and ankle surgery was substantial, which confirms that the interobserver reliability increases with subspecialty training. Therefore, Mansoura classification for foot and ankle Charcot has acceptable reliability and could be promising in the evaluation and guiding the management of such cases, especially among foot and ankle surgeons.

## Data Availability

The datasets used and/or analysed during the current study are available from the corresponding author on reasonable request.
